# The Addiction Neurocircuitry and Resting‐State Functional Connectivity in Cannabis Use Disorder: An fMRI Study

**DOI:** 10.1111/adb.70105

**Published:** 2025-12-03

**Authors:** Hannah Thomson, Izelle Labuschagne, Arush Honnedevasthana Arun, Eugene McTavish, Hannah Sehl, Adam Clemente, Emillie Beyer, Marianna Quinones‐Valera, Peter Rendell, Gill Terrett, Lisa‐Marie Greenwood, Govinda Poudel, Victoria Manning, Chao Suo, Valentina Lorenzetti

**Affiliations:** ^1^ Neuroscience of Addiction and Mental Health Program, Healthy Brain and Mind Research Centre, School of Behavioural and Health Sciences, Faculty of Health Sciences Australian Catholic University Fitzroy Victoria Australia; ^2^ School of Behavioural and Health Sciences Australian Catholic University Fitzroy Victoria Australia; ^3^ School of Psychology University of Queensland Brisbane Queensland Australia; ^4^ School of Medicine and Psychology, Australian National University College of Health and Medicine Australian National University Canberra Australia; ^5^ Mary MacKillop Institute for Health Research Australian Catholic University Melbourne Victoria Australia; ^6^ Braincast Neurotechnologies Melbourne Victoria Australia; ^7^ Turning Point, Eastern Health Monash University Melbourne Victoria Australia; ^8^ Turner Institute for Brain and Mental Health, School of Psychological Sciences Monash University Clayton Victoria Australia

**Keywords:** addiction, cannabis dependence, CUD, functional magnetic resonance imaging, region of interest, rsFC, seed‐based connectivity

## Abstract

Cannabis use disorder (CUD) affects ~22‐million people globally and is characterised by difficulties in cutting down and quitting use, but the underlying neurobiology remains unclear. We examined resting‐state functional connectivity (rsFC) between regions of interest (ROIs) of the addiction neurocircuitry and the rest of the brain in 65 individuals with moderate‐to‐severe CUD who reported attempts to cut down or quit, compared to 42 controls, and explored the association between rsFC and cannabis exposure and related problems, to elucidate potential drivers of rsFC alterations. The CUD group showed greater rsFC than controls between ROIs implicated in reward processing and habitual substance use (i.e., nucleus accumbens, putamen and pallidum) and occipito/parietal areas implicated in salience processing and disinhibition. Putamen‐occipital rsFC correlated with levels of problematic cannabis use and depression symptoms. CUD appears to show neuroadaptations of the addiction neurocircuitry, previously demonstrated in other substance use disorders.

## Graphical abstract

Resting‐state functional connectivity was compared between 65 individuals with moderate‐to‐severe cannabis use disorder (CUD) and 42 controls. The CUD group showed greater rsFC between reward‐ and habit‐related regions (nucleus accumbens, putamen, pallidum) and occipito‐parietal areas involved in salience and disinhibition. Putamen–occipital rsFC correlated with problematic cannabis use and depression. Findings suggest neuroadaptations within addiction‐relevant circuitry.

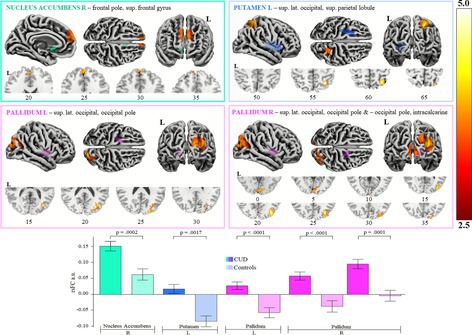

## 1 Introduction

Cannabis use disorder (CUD) is experienced by ~22 million individuals globally [1] and can be characterised by an inability to voluntarily cease or reduce consumption of cannabis despite experiencing physical or psychological harms [2], for example, risk‐taking behaviours, including driving under the influence [3]. CUD is associated with a greater prevalence of mental health disorders such as depression, anxiety and psychosis [4, 5] and reduced performance on cognitive tasks [6, 7]. Psychosocial problems associated with CUD have been partly ascribed to neurobiological changes within the addiction neurocircuitry [8]. Of relevance, recent functional neuroimaging methodologies have measured the functional integrity of the addiction neurocircuitry in vivo, minimising confounding effects of task‐related cognitive processes associated with task performance, for example, greater cognitive effort and the involvement of task‐specific cognitive processes [9]. One such methodology is resting‐state functional connectivity (rsFC). rsFC measures correlations between the spontaneous fluctuations of the blood oxygen level‐dependent (BOLD) signal of two or more spatially distinct brain regions, whereas people are at rest and without performing cognitively demanding tasks [9, 10]. Investigating rsFC in CUD can be valuable to offer novel insights into how the integrity of the addiction neurocircuitry is affected during rest and when the system is not challenged by meeting the demands of specific cognitive tasks.

Mounting evidence from functional magnetic resonance imaging (fMRI) studies demonstrates that people who regularly use cannabis show different brain function during rest (i.e., rsFC)—within brain pathways of the addiction neurocircuitry [8]. Compared to controls, they show greater rsFC between frontal regions and other frontal regions (e.g., anterior cingulate cortex [ACC], prefrontal cortex [PFC] and orbitofrontal cortex [OFC]), temporal regions (e.g., hippocampus and amygdala) and striatal regions (e.g., nucleus accumbens [NAc], putamen, pallidum and caudate) [11]. Researchers have proposed that identified changes may involve circuitry important for cognitive functions commonly altered in cannabis use disorder, for example, greater salience of cannabis rewards, lower sensitivity to rewards other than cannabis, disinhibition and stress [11]. There is also preliminary evidence that rsFC changes in cannabis users correlated with cannabis exposure metrics (i.e., age of use onset and duration) [11]; however, the significance and direction of the findings have been inconsistent and require further investigation.

The evidence on rsFC changes in cannabis users is limited by methodological issues. First, only two fMRI rsFC studies, to the best of our knowledge, have measured if their participants endorsed DSM‐5 criteria for CUD [2, 12, 13]. In both studies, rsFC significantly differed between CUD and control groups and correlated with metrics of cannabis use (Structured Clinical Interview of DSM‐5–research version [SCID‐5‐RV] scores, Cannabis Use Disorders Identification Test–Revised [CUDIT‐R] scores and cannabis grammes per week), supporting the importance of considering both CUD status and levels of cannabis use. However, the majority of literature in this field does not account for CUD status and has not formally selected participants with more severe forms of CUD with failed attempts to cut down or quit; hence, the extant literature's relevance for people with a CUD remains unclear [11]. This issue is important as a substantial number of individuals who use cannabis report failed attempts to quit [14], and the underlying neurobiology remains unexamined. Second, the impact of confounding variables that can affect brain function independently or in interaction with CUD has been inconsistently examined, including key sociodemographic data (e.g., age and sex), alcohol and nicotine consumption and mental health symptoms (e.g., depression) [11]. Third, cannabis exposure and related problems have seldom been measured in relation to rsFC. Therefore, it remains unclear if these factors play a role in driving altered rsFC reported in cannabis users (e.g., severity of CUD, dosage of recent cannabis use, urine levels of 11‐nor‐9‐carboxy‐Δ^9^‐tetrahydrocannabinol: creatinine [THC‐COOH: creatinine ng/mL] and duration of cannabis use and of abstinence). Finally, about half of the extant literature consists of small sample sizes (i.e., *n* < 25) [11] and may be statistically underpowered to detect subtle rsFC alterations.

We aimed to overcome these limitations by examining rsFC in a sample of 107 participants (35 females and 72 males) aged 18–56. The primary aim was to examine rsFC in 65 non‐intoxicated individuals with a moderate‐to‐severe CUD who had recently tried to cut down/cease cannabis use, compared to 42 controls, accounting for age, sex and standard drinks in the previous 30 days. It was hypothesised that compared to controls, the CUD group would show different rsFC between regions of interest (ROIs) and the rest of the brain, utilising a seed‐to‐whole brain analysis. ROIs were concurrently integral to the addiction neurocircuitry [8] and with known rsFC alterations in cannabis users [11]. Said ROIs were also dense in cannabinoid receptors type 1 (CB_1_R), to which delta‐9‐tetrahydrocannabinol (THC), the main psychoactive compound of cannabis binds [15, 16]. The selected ROIs were in the striatum (i.e., NAc, putamen and caudate), basal ganglia (pallidum), medial temporal regions (i.e., hippocampus and amygdala) and the ACC. By targeting these specific regions within the addiction neurocircuitry and investigating their connectivity with the rest of the brain, the findings aim to enhance our understanding of the neurobiological underpinnings of CUD.

The secondary aim was to explore how significantly altered rsFC in the cannabis group correlated with metrics of cannabis exposure and related problems, including SCID‐5‐RV scores, CUDIT‐R scores, grammes/past 30 days, urine levels of THC‐COOH: creatinine ng/mL, hours since last use, age of onset, duration of regular use, depression symptom scores and nicotine dependence [12, 13, 17, 18, 19]. By examining these relationships, the study aims to uncover potential drivers of rsFC changes, offering deeper insights into the neurobiological mechanisms of CUD.

## 2 Methods

### 2.1 Study Design

This cross‐sectional, case–control study was nested within a larger, preregistered project (ISRCTN ID: 76056942) and was approved by the Australian Catholic University Human Research and Ethics Committee (HREC:2019‐71H).

### 2.2 Participants

#### 2.2.1 Recruitment

All participants were recruited from the Greater Melbourne Metropolitan area via community‐based flyers and online advertisements, which directed them to an online screening survey. Participants were further screened via phone call to confirm eligibility for the study.

#### 2.2.2 Key Inclusion and Exclusion Criteria

Participants aged 18–55 (one participant who turned 56 between screening and testing was retained in the sample) were eligible if they either endorsed a moderate‐to‐severe CUD [2], with use of cannabis on a daily or almost daily basis for ≥ 12 months and with at least one attempt to quit or reduce cannabis use within the past 24 months, or were controls who did not endorse the use of cannabis at any stage in the 12 months prior to testing or > 50 lifetime uses of cannabis. A complete and detailed overview of the inclusion and exclusion criteria is displayed in the Supporting Information.

### 2.3 Procedure

Participants provided written informed consent before completing a ~4‐ to 6‐h session that included demographic surveys, fMRI scanning, behavioural and cognitive assessments, and urine sample collection. The sessions were conducted by experienced and specially trained researchers and students at the Monash Biomedical Imaging facility in Clayton, Victoria. Please see the Supporting Information for detailed participant procedures and testing measures, which were described in detail in the study preregistration (https://doi.org/10.1186/ISRCTN76056942).

### 2.4 fMRI Acquisition and Analysis

**FIGURE 1:**
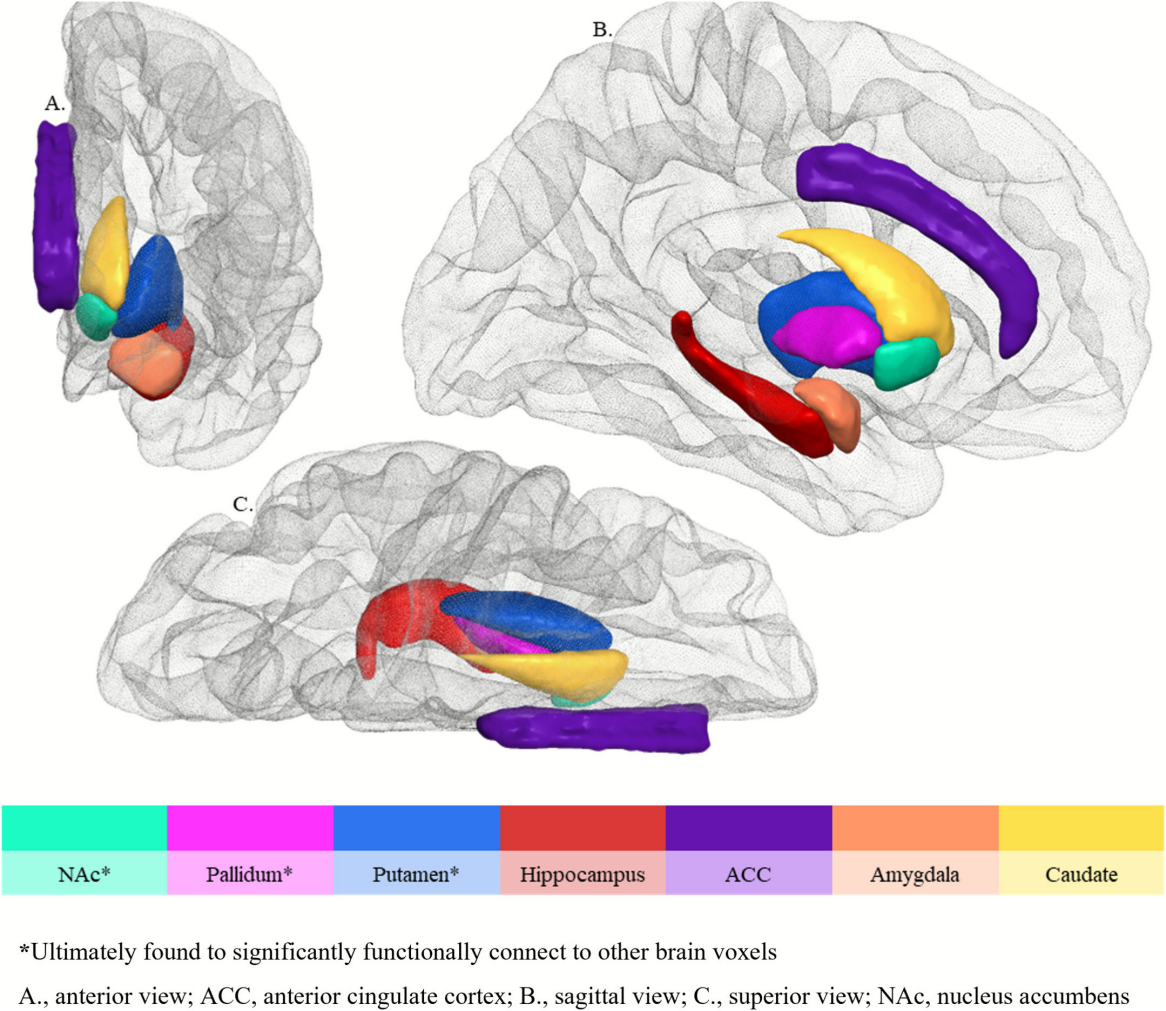
Overview of examined seeds, shown here in the left hemisphere only. *Ultimately found to significantly functionally connect to other brain voxels. (A) Anterior view; ACC, anterior cingulate cortex. (B) Sagittal view. (C) Superior view; NAc, nucleus accumbens.

MRI data acquisition, preprocessing, quality control and analysis are detailed in the Supporting Information. Key acquisition parameters were as follows: TE = 2.07 ms, TR = 2300 ms, flip angle = 9°, 192 sagittal slices without gap, field of view 256 × 256 mm, yielding a 1 × 1 × 1 mm resolution, with a total acquisition time of 5 min. Resting‐state fMRI scans (189 volumes) were acquired over 8 min using T2* weighted Echo Planar Imaging (EPI) with TR = 2500 ms, TE = 30 ms, flip angle = 90°, field of view = 192 mm, matrix = 64, 44 slices without gap and a voxel size of 3 mm^3^. Standard preprocessing pipelines were conducted using CONN toolbox 20.b [20] on a cloud‐based computational platform MASSIVE [21]. A priori brain ROIs were selected as seeds: NAc, putamen, pallidum, caudate, hippocampus, amygdala and the ACC. Figure 1 overviews the seeds examined. Functional connectivity maps for each seed were generated for each individual and fed into the next statistical analysis.

### 2.5 Statistical Analyses

#### 2.5.1 Sample Characteristics

Groups were compared using Chi‐squares for categorical variables (i.e., sex and handedness), *t* tests for normally distributed scalar data (i.e., FSIQ and perceived stress) and Mann–Whitney *U* tests for other nonnormally distributed scalar data.

#### 2.5.2 Seed‐to‐Whole Brain Functional Connectivity Analysis

Statistical analysis was conducted using the same CONN toolbox to examine group differences for seed‐to‐whole brain connectivity, adjusting for age, sex and past 30‐day standard drinks. Two corrections for multiple comparisons were applied before significant ROIs were reported. Specifically, a false discovery rate (FDR) correction was applied to control for voxel‐wise multiple comparison errors, and a Bonferroni correction was applied for independent seed selections. Please refer to Supporting Information for details.

#### 2.5.3 Brain‐Behaviour Correlations

The rsFC values within each significant ROI were extracted at the individual level. Spearman's correlations were examined for the relationship between post hoc ROI values, and cannabis exposure and related problems: number of DSM‐5 CUD symptoms, CUDIT‐R scores, cannabis grammes/past 30 days, THC‐COOH: creatinine ng/mL in urine, hours since last cannabis use, age of onset/duration of regular cannabis use, depression symptoms and FTND. Correlations' *p* values were Bonferroni‐corrected to *p* < 0.001 after dividing *p* < 0.05 by 45 analyses run. All descriptive statistics and brain‐behaviour correlations were run via SPSS Version 28.

## 3 Results

### 3.1 Sample Characteristics

Sample characteristics are outlined in Table 1. The sample included 107 participants (65 with moderate‐to‐severe CUD and 42 controls) aged 18–56 years (mean = 27, 35 females and 72 males). Groups were matched by sex and age. The CUD and control groups did not significantly differ for years of education, FSIQ estimates and handedness, as well as for several mental health symptom scores: state anxiety, perceived stress and COVID stress.

**TABLE 1:** Sample characteristics and group differences for sociodemographic data, FSIQ estimate, substance use and related problems, and mental health symptom scores.

Variable	Moderate‐to‐severe CUD	Controls	Group difference		
			*Z*/*t*/*χ* ^*DF*^		*p* value
*N* total (female)	65 [19]	42 [16]	*χ*	0.91^1^	0.340
Right‐handed *N*	61	36	*χ*	0.09^1^	0.762
	Mean (SD)				
Age, years	27 (8)	28 (9)	*Z*	0.21	0.835
Education, years	15 (3)	16 (4)	*Z*	0.80	0.426
FSIQ estimate	107 (10)	109 (13)	*t*	0.80^60^	0.426^a^
BDI‐II	11 (8)	6 (8)	*Z*	4.09	< 0.001***
STAI‐Y (state)	33 (9)	30 (8)	*Z*	1.70	0.090
PSS	16 (8)	13 (7)	*t*	1.70^103^	0.093
COVID Stress	11 (16)	10 (9)	*Z*	0.68	0.496
CAPE					
Positive psychotic experiences	39 (12)	31 (9)	*Z*	3.58	< 0.001***
Depressive symptoms	23 (9)	19 (8)	*Z*	2.39	0.017*
Negative psychotic experiences	40 (14)	32 (13)	*Z*	2.75	0.006**
Alcohol					
Days/past 30 days	6 (7)	3 (4)	*Z*	3.06	0.002**
Drinks/past 30 days	30 (46)	11 (19)	*Z*	3.21	0.001**
AUDIT	7 (4)	3 (3)	*Z*	4.80	< 0.001***
Cigarette					
Days/past 30 days	10 (13)	0 (−)		—	—
Dose/past 30 days	64 (127)	0 (−)		—	—
FTND	1 (2)	0 (−)		—	—
Cannabis exposure	Mean (SD)				
CUD symptoms	7 (2)	—		—	—
CUDIT‐R	16 (5)	—		—	—
Days/past 30 days	26 (5)	—		—	—
Dosage (grammes)					
Past 30 days	27 (20)	—		—	—
Lifetime	2296 (3448)	—		—	—
Past year	324 (266)	—		—	—
Duration, years	7 (8)	—		—	—
Age					
First use, years	17 (3)	—		—	—
Regular use, years	20 (6)	—		—	—
Urine THC‐COOH					
Creatinine, ng/mL	229 (236)	—		—	—
Abstinence, hours	21 (12)	—		—	—

*Note:* Bolded *p* values represent statistically significant differences: *< 0.05; **< 0.01; ***< 0.001.

Abbreviations: *χ*, Chi‐square; AUDIT, Alcohol Use Disorder Identification Test; BDI‐II, Beck Depression Inventory—second edition; CAPE, Community Assessment of Psychic Experiences; CUD, cannabis use disorder; CUDIT‐R, Cannabis Use Disorder Identification Test—Revised; DF, degrees of freedom; FSIQ, Full Scale Intelligence Quotient; FTND, Fagerström Test for Nicotine Dependence; *N*, sample size; ng/mL, nanogrammes per millilitre; PSS, Perceived Stress Scale; SD, standard deviation; STAI‐Y (state), State–Trait Anxiety Index—Y Form (state subscale); *t*, independent sample *t* test; THC‐COOH: creatinine, 11‐nor‐9‐carboxy‐Δ^9^‐tetrahydrocannabinol: creatinine; *Z*, Mann–Whitney *U*.

^a^ Homogeneity of variance not assumed.

The CUD group had significantly higher scores than controls for depression symptoms and all CAPE subscales, as well as all alcohol use metrics: alcohol use days/past 30 days, standard drinks/past 30 days and AUDIT scores.

There were 28 of 65 people with CUD who endorsed nicotine use over the past 30 days; they scored a mean of 2 on the FTND, indicating ‘very low’ nicotine dependence overall. No controls reported past 30‐day nicotine use.

### 3.2 Levels of Cannabis Exposure and Related Problems

All people in the CUD group met the criteria for a moderate‐to‐severe CUD; on average, participants had a severe CUD, corroborated by DSM‐5 CUD symptoms. Participants used cannabis almost daily over the past 30 days and about a gramme per day.

All people with a CUD reported having attempted to cut down or reduce their cannabis use at least once over the past 2 years and used cannabis at least 4 days per week for a minimum of 12 months. The mean age of first cannabis use was 17, and the mean age of regular use was 20.

The CUD group abstained from cannabis for a mean of 21 h before testing. The presence of THC metabolites in urine confirmed cannabis use in the CUD group, accounting for variable hydration status.

### 3.3 Group Differences in Resting‐State Functional Connectivity

**FIGURE 2:**
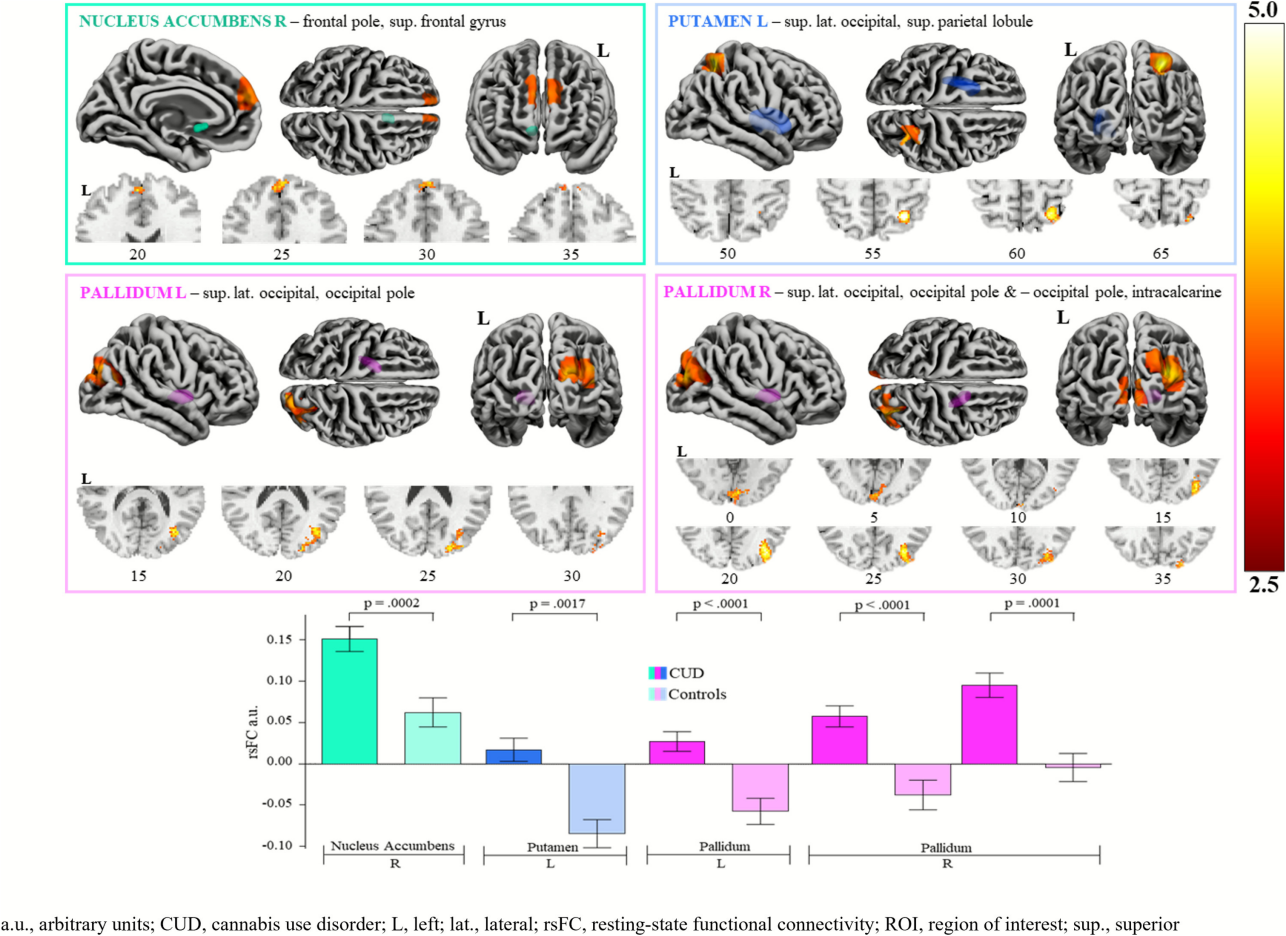
Significantly greater resting‐state functional connectivity in people with a cannabis use disorder compared to controls, between ROI seeds and clusters. a.u., arbitrary units; CUD, cannabis use disorder; L, left; lat., lateral; rsFC, resting state functional connectivity; ROI, region of interest; sup., superior.

As shown in Table 2 and Figure 2, the CUD group had higher rsFC than controls between fronto‐striatal, occipito‐parieto‐striatal and occipito‐basal ganglia region pairings, accounting for age, sex and past 30‐day standard drinks, with false discovery rate (FDR) and Benjamini–Hochberg (1995) corrections applied. Specific region pairings showing greater rsFC were NAc‐frontal pole/SFG, putamen‐occipito/parietal and pallidum‐occipital/intracalcarine cortex.

**TABLE 2:** Overview of significantly greater resting‐state functional connectivity in people with a CUD compared to controls, between ROI seeds and their respective clusters.

Seed		Cluster (specific regions)	Peak (*x*, *y*, *z*)	*K*	Size p‐FDR	B‐H adjusted *α*
NAc	Right	Frontal pole bilateral, SFG left	6, 54, 22	190	0.0002***	0.0133
Putamen	Left	Superior lateral occipital, superior parietal lobule right	30, −56, 56	178	0.0017**	0.0167
Pallidum	Left	Superior lateral occipital, occipital pole right	36, −74, 18	317	< 0.0001***	0.0067
	Right	Superior lateral occipital, occipital pole right	34, −76, 24	501	< 0.0001***	0.0033
		Occipital pole, intracalcarine bilateral	4, −90, 2	229	0.0001***	0.0100

*Note:* Bolded *p* values represent statistically significant pairings: **< 0.01; ***< 0.001.

Abbreviations: B‐H, Benjamini–Hochberg; FDR, false discovery rate; *K*, number of voxels; NAc, nucleus accumbens; SFG, superior frontal gyrus.

### 3.4 Brain‐Behaviour Correlations

**FIGURE 3:**
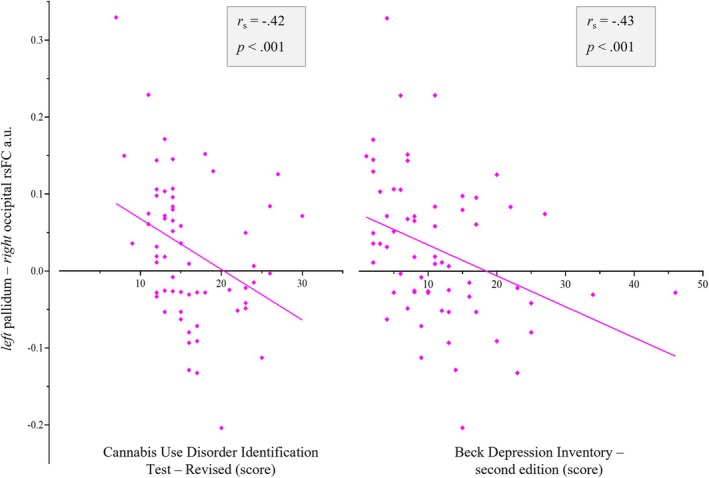
Spearman's rho (*r*_*s*_) correlations within the CUD group, between rsFC pairs showing group differences (rsFC beta values) significantly correlated with metrics of cannabis exposure and related problems and depression scores, using Bonferroni adjusted alpha threshold of *p* < 0.001 (0.05 ÷ 45).

Significant correlations were observed between greater left pallidum–occipital cortex rsFC and lower CUDIT‐R scores (*p* = 0.0007) and lower depression symptom scores (i.e., BDI‐II, *p* = 0.0004), following the application of a Bonferroni correction (Figure 3). No other correlations reached statistical significance (see Table S1).

## 4 Discussion

To our knowledge, this is the first resting‐state functional connectivity (rsFC) fMRI study to examine individuals with moderate‐to‐severe CUD who have attempted to cut down or cease cannabis use, while controlling for age, sex and recent alcohol use. Consistent with prominent neuroscientific theories of addiction, CUD was shown to be associated with altered rsFC across striatal and basal ganglia subcortical regions and frontal, occipital and parietal cortical regions compared to controls. Identified regions have been implicated in reward processing, disinhibition and habitual/compulsive substance use (i.e., NAc‐frontal pole/SFG, putamen‐occipito/parietal cortex and pallidum‐occipital/intracalcarine cortex), some of which correlated with the severity of CUD scores and depression symptom scores.

The current study consistently observed greater rsFC in CUD compared to controls, particularly within fronto‐striatal and occipito‐parieto‐striatal networks. The location and direction of the findings are in line with previous work reporting greater rsFC in overlapping networks in people who regularly use cannabis compared to controls (i.e., greater NAc‐ACC cluster rsFC) [22]. The direction of rsFC change may fluctuate as a function of the intoxication cycle. Indeed, greater rsFC has been consistently reported when participants are nonintoxicated [23], whereas lower rsFC has been consistently reported during acute cannabis intoxication [24]. Therefore, chronic intoxication with THC over time may lead to greater connectivity of mesocorticolimbic regions (e.g., between frontal regions and the motor cortex, insula and temporo‐parietal regions) [23].

We found greater putamen‐occipito/parietal and pallidum‐occipital/intracalcarine rsFC in CUD compared to the control group. This pattern of rsFC may underlie habitual cannabis use and altered salience processing observed in CUD [25]. Indeed, both the putamen and pallidum have been implicated in cognitive processes reportedly altered in regular cannabis users, including habit formation/automated behaviour [25] and habitual substance use [25].

The observed correlation between greater pallidum‐occipital cortex rsFC and lower CUDIT‐R scores contributes to the literature, which suggests that rsFC changes may reflect neural adaptations linked to cannabis use severity [12]. These findings align with previous evidence implicating the pallidum and putamen in habit formation and compulsive substance use [25]. This notion is partially supported by the association identified herein between pallidum‐occipital cortex rsFC and CUDIT‐R scores. Similarly, greater activation in striatal and basal ganglia regions has emerged in cannabis users with greater severity of cannabis‐related problems, frequency of use and/or earlier age of onset across task‐based ‘cue‐reactivity’ fMRI research [26]. In contrast, task‐based fMRI studies have also detected decreased activity in the putamen and in occipital regions, in long‐term cannabis users [27]. Occipital (and some parietal) regions support attentional processing/direction towards salient information such as ‘uniquely coloured lines’, or specific‐coloured circles, presented in the participant's visual field [28], which could reflect neuroadaptations in attention/thoughts towards cannabis/salience pathways that persist during rest.

The notion that established rsFC alterations in a cannabis‐using group may be driven in part by the presence/severity of CUD [29] is supported by the negative association identified between pallidum‐occipital cortex rsFC and CUDIT‐R scores. Alterations in such neural structures are thought to underlie emotional processing, habit formation and reward‐based learning. Additionally, this correlation was largely aligned with correlations reported by Aloi et al. [12], who demonstrated a negative association between CUDIT‐R scores and amygdala‐occipital (cuneus) rsFC. Negative correlations between CUDIT‐R scores and rsFC have also been reported for the following pairings: crus‐brain stem and crus‐cerebellar lobule [30] and amygdala‐paracentral/supplementary motor area and amygdala‐temporal pole [12]. Additionally, negative associations established between pallidum‐occipital cortex rsFC and depression scores were also somewhat in keeping with previous research [31, 32]. Depression scores were previously shown to positively correlate with rsFC between frontal regions [31] and between frontal and parietal regions [32]. These findings highlight the importance of controlling for mood‐related symptomology when examining rsFC in cannabis‐using populations. It also suggests that people with comorbidities (i.e., mood disorder) may experience additional neuroadaptations and hence may be an especially vulnerable subgroup of people with a CUD.

We reported greater fronto‐striatal (NAc‐frontal pole/SFG) rsFC in people with a CUD, which may reflect increased engagement of salience pathways sensitive to THC exposure [33]. Indeed, THC increases dopamine synthesis within the NAc [34, 35], which might subsequently affect the function of the NAc, and possibly of interconnected frontal pathways implicated in salience processing. It is acknowledged that we cannot ascertain which region drives the connection or if both/all regions activate in unison, from the identified rsFC alterations. Nevertheless, increased fronto‐striatal rsFC may be a key contributor to cannabinoid reinforcement [36]. Indeed, neural projections from the NAc to the PFC may mediate the experience of ‘wanting’ cannabis and urges to use cannabis [33]. In line with this notion, the NAc plays a key role in assigning value to stimuli [37], whereas frontal regions have been linked to a loss of control over substance use [38]. Therefore, fronto‐striatal regions may be more prominently implicated in more severe forms of CUD, where a loss of control over cannabis use can be a key feature.

Overall, our finding of greater fronto‐striatal connectivity in people with more severe forms of CUD is in keeping with rsFC fMRI literature in cannabis users more broadly (for a review, see Thomson et al. [11]) and in studies in other substance use disorders (SUDs). Specifically, greater frontostriatal rsFC was observed in current, ‘chronic’ cannabis users [17], in cannabis dependent participants who abstained from cannabis for 28 days [22, 39], and in users of different substances, for example, cocaine amongst others [40]. Taken together, the findings suggest that fronto‐striatal rsFC alterations may underpin CUD, although the time course of the changes is unclear as they may predate or follow CUD (or both) and may persist following abstinence from cannabis.

To the authors' knowledge at the time of writing, this is the first fMRI study to examine rsFC in current cannabis users with moderate‐to‐severe CUD who report failed attempts to cut down or quit their consumption. Findings herein lend support for the first time that the neurocircuitry of CUD overlaps with the addiction neurocircuitry implicated in reward/salience processing and compulsive use, as postulated by prominent neuroscientific theories [33, 41], which have largely been based on substances other than cannabis (e.g., cocaine, alcohol and opioids) [41]. Additionally, up until now, established neuroscientific theories of addiction have been largely validated in outdated diagnostic systems (e.g., DSM‐IV), which do not reflect the current diagnostic system relying on the DSM‐5 [42]. Furthermore, we have specifically examined cannabis users to endorse an inability to reduce or cease their use, extending our finals to a larger subset of individuals with CUD [14]. Longitudinal neuroimaging studies (e.g., Adolescent Brain Cognitive Development [ABCD] study; https://abcdstudy.org/) are required to unpack the time course of rsFC in people who develop a CUD. If indeed such changes did predate CUD‐onset, patterns of rsFC alterations identified here could contribute to the mapping of biomarkers for CUD, to identify at‐risk populations in future research, but could challenge/undermine existing evidence for fMRI differences in CUD relative to control groups.

A strength of this study is its robust methodology, including a focus on a well‐defined sample of individuals with moderate‐to‐severe CUD who have attempted to reduce or cease use. By controlling for confounding factors such as age, sex and alcohol use, the findings provide a clearer understanding of the specific neural alterations associated with CUD. First, the CUD and control groups did not significantly differ in many sociodemographic and mental health‐related variables that can also affect rsFC (e.g., age, anxiety, gender, handedness and stress). Second, confounders that showed significant group differences (i.e., past‐30‐day alcohol exposure) and variables that exert a major influence on brain function (i.e., age and sex) were controlled for in all rsFC analyses, minimising their influence on results. Third, participants abstained from cannabis use for at least 12 h prior to scanning; neither ‘duration of abstinence’ nor ‘THC‐COOH: creatinine ng/mL’ correlated with rsFC alterations in the CUD group. Therefore, it was unlikely that acute cannabis effects (i.e., intoxication) confounded results. As well as controlling for potential confounders, another strength of the study was the recruitment of heterogenous participants with a range of depression, anxiety, psychiatric symptoms scores, alcohol and nicotine use, which is representative of a population with CUD [43].

Regarding sample size, it has been posited that brain‐behaviour correlations are adequately replicable in samples ranging from 20 participants [44] to 42 participants [45]. Thus, robust neuroimaging studies with targeted samples with a CUD, such as the current experiment, are required to advance the understanding of the neurobiology of CUD. In the future, multisite consortia studies could be utilised to substantially increase power and demonstrate replicability of findings (e.g., ABCD and the ENIGMA Addiction working group; www.enigmaaddictionconsortium.com).

The altered brain networks in CUD identified herein, particularly pallidum‐occipital cortex rsFC associated with CUD severity, could be targeted by brain‐based interventions for people aiming to gain increased control over, or reduce, their cannabis use. This includes neurofeedback [46], Transcranial Magnetic Stimulation (TMS) [47] and mindfulness‐based interventions [48], known to reduce alterations of these pathways in SUD. Neurofeedback, which entails the provision of ‘real time’ feedback during an fMRI scan, regarding brain activation under certain conditions (established previously during substance cravings for alcohol, tobacco and cocaine [49]), allows for the development of personalised neural targets, which the participant can be trained to self‐regulate [50]. rsFC alterations identified herein could be used to guide neural neurofeedback targets. Furthermore, mindfulness‐based interventions have been linked to alterations of rsFC between brain regions overlapping those implicated in this manuscript and to a reduction in cigarette smoking [48]. Future research could examine if mindfulness interventions targeting core features of CUD mitigate its neural circuitry.

This study is the first fMRI study to examine rsFC in selected ROIs of individuals with moderate‐to‐severe CUD versus controls, with a robust rationale for the ROIs. We demonstrated greater rsFC across subcortical (striatal and basal ganglia) and cortical regions (frontal, occipital and parietal). The changes were partly correlated with the severity of CUD, and they may reflect neuroadaptations in salience pathways that follow the neuroadaptations within the addiction neurocircuitry. Such adaptations in nonintoxicated individuals with CUD may follow documented THC‐induced reductions in rsFC and dopamine release within the NAc [24]—a hypothesis that needs corroboration by experimental studies in CUD before, during and after THC intoxication.

It was thought that greater putamen/pallidum‐occipital/occipito‐parietal rsFC may underlie habitual cannabis use and altered salience processing observed in CUD, in part driven by CUD severity. Furthermore, fronto‐striatal rsFC increases in the CUD group may reflect the engagement of salience pathways sensitive to THC exposure, secondary to the effect of THC on dopamine synthesis within the NAc. Longitudinal neuroimaging studies are required to confirm if identified changes predate or follow CUD (or both), and multisite consortia studies could be instrumental in replicating the findings in individuals with varying CUD severity and in cannabis users without a CUD. These findings lend support to prominent neuroscientific theories of addiction insofar as their application to CUD and are likely representative of cannabis‐using populations with more severe forms of CUD and comorbid depression and anxiety. Future research could utilise the results herein to develop brain‐based interventions for people aiming to change their cannabis use, such as neurofeedback and mindfulness‐based intervention.

## Author Contributions

All authors edited the manuscript. A.H.A. contributed to fMRI quality checks and analyses, with general direction on the technical aspects from C.S. and G.P. and on the theoretical aspects from V.L. A.C. managed all the operations of the study. L.‐M.G. provided high‐level and ongoing input on all aspects of the study. I.L. supported the setup of the study protocol. V.L. designed and led the study as C.I., supervised all students and staff involved and led all revisions. V.M. provided high‐level and ongoing input on the design and conduct of the study and edited the first full draft of the manuscript as well as subsequent drafts. E.M. contributed to statistical analyses of the behavioural data and provided input on theoretical aspects of the manuscript. G.P. provided high‐level and ongoing input on the design and conduct of the study and edited the first full draft of the manuscript as well as subsequent drafts. M.Q.‐V. supported data collection. E.B. supported data collection. P.R. supported the setup of the study protocol. H.S. supported the study setup and data collection. C.S. provided high‐level and ongoing input on all aspects of the study with a focus on neuroimaging/statistical analysis and data visualisation. G.T. supported the setup of the study protocol. H.T., under the PhD supervision of V.L., C.S. and I.L., developed the theoretical framework of the manuscript, conducted fMRI quality checks, contributed to the neuroimaging data analysis, drove statistical behavioural analysis, created the first draft and integrated subsequent revisions.

## Funding

Valentina Lorenzetti was supported by an Al and Val Rosenstrauss Research Fellowship (2022–2026) and by a National Health and Medical Research Council Investigator Grant (2023–2027, ID 2016833) and an Australian Catholic University competitive scheme.

The work within the Neuroscience of Addiction and Mental Health Program, Healthy Brain and Mind Research Centre was supported via an ACU competitive scheme. Hannah Thomson and Hannah Sehl were funded by Australian Government Research Training Program (RTP) Stipend scholarships. Victoria Manning has received funding from the National Health and Medical Research Council (NHMRC), VicHealth, Department of Health Victoria; the Victorian Responsible Gambling Foundation; the National Centre for Clinical Research on Emerging Drugs (NCCRED), HCF Research Foundation and philanthropic organisations. This study was supported by the Rebecca L. Cooper Medical Research Foundation (F20221117).

## Disclosure

Hannah Thomson presented these results via poster presentation on 25 July 2022 at the British Association for Psychopharmacology, 2022 London Summer Meeting.

## Ethics Statement

This cross‐sectional, case–control study was nested within a larger, pre‐registered project (ISRCTN ID: 76056942, https://doi.org/10.1186/ISRCTN76056942) and was approved by the Australian Catholic University Human Research and Ethics Committee (HREC:2019‐71H).

## Conflicts of Interest

Izelle Labuschagne is the founder and director of Complete Thesis Support, which provides developmental programmes for research students. Victoria Manning was the founder, CEO, director and a shareholder of Cognitive Training Solutions Pty Ltd. between March 2021 and Aug 2023, which commercialised the SWiPE app that delivers Cognitive Bias Modification to reduce alcohol use. Govinda Poudel is the founder, director and CTO of BrainCast Pty Ltd., which has developed novel brain imaging markers for monitoring brain injury. Hannah Thomson contracts for Syneos Health Learning Solutions, with the Insights and Evidence Generation Team in Patient Insights and Assessment Research (Implementation Science). The rest of the authors declare no conflicts of interest.

## Supporting information


**Table S1:** CUD group correlations between rsFC pairs showing group differences (rsFC beta values) correlated with metrics of cannabis exposure and related problems, depression scores, and nicotine dependency, using Bonferroni adjusted alpha threshold of *p* < 0.001 (0.05 ÷ 45).
**Figure S1:** Monash Biomedical Imaging Magnetic Resonance Imaging (MRI) Screening and Information Form.

## Data Availability

All data are available upon reasonable request from Dr. Valentina Lorenzetti (valentina.lorenzetti@acu.edu.au), principal investigator. Data will include relevant group allocations and outcome variables and will be anonymised. Data will be available either as they are published or on request. A time limit will not be set on the duration of availability. Data will be shared with anyone who wishes to access them, for meta‐analyses or other preapproved purposes, via email. All participants provided informed consent. All data are deidentified.
