# Supplementary material for: The Addiction Neurocircuitry and Resting‐State Functional Connectivity in Cannabis Use Disorder: An fMRI Study
Source: Addict Biol. 2025 Dec 3;30(12):e70105. doi: 10.1111/adb.70105 (PMC12675133; doi:10.1111/adb.70105)
Supplement: Supplementary file 1 — Table S1: CUD group correlations between rsFC pairs showing group differences (rsFC beta values) correlated with metrics of cannabis exposure and related problems, depression scores, and nicotine dependency, using Bonferroni adjusted alpha threshold of p < 0.001 (0.05 ÷ 45). Figure S1: Monash Biomedical Imaging Magnetic Resonance Imaging (MRI) Screening and Information Form. [file ADB-30-e70105-s001.docx]

**SUPPLEMENTARY INFORMATION**

**TABLE OF CONTENTS**

Methods

1. INCLUSION AND EXCLUSION CRITERIA - 2 -
2. PROCEDURE - 3 -
3. MEASURES - 4 -
4. fMRI ACQUISITION AND PROCESSING - 5 -

Tables / Figures

1. SUPPLEMENTARY TABLE 1. **CUD group correlations** - 8 - **between rsFC pairs showing group differences (rsFC
   beta values) correlated with metrics of cannabis exposure
   and related problems, depression scores, and nicotine
   dependency, using Bonferroni adjusted alpha threshold
   of p<.001 (.05 ÷ 45)**
2. SUPPLEMENTARY FIGURE 1. **Monash Biomedical** - 9 - **Imaging Magnetic Resonance Imaging (MRI) Screening
   and Information Form**

References - 10 -

Methods

1. INCLUSION AND EXCLUSION CRITERIA

Unless otherwise specified, inclusion and exclusion criteria were confirmed by participant self-report and the use of a comprehensive online screening survey followed by detailed and focused screening over the phone.

Inclusion Criteria

Inclusion criteria for all participants were: (1) age between 18 years and 55 years; (2) having normal-to-corrected vision; (3) fluency in English; and (4) ability to attend testing sessions in person.

For people with a CUD only: (1) use of cannabis on a daily or almost daily basis for ≥ 12 months; (2) an attempt to quit or to reduce cannabis use at least once within the past 24 months; and (3) a diagnosis of moderate-to-severe CUD, confirmed by the endorsement of ≥ 4 CUD symptoms from the DSM-5 (American Psychiatric Association [APA], 2013), measured using the Structured Clinical Interview of DSM-5 – research version (SCID-5-RV; First et al., 2015).

Exclusion Criteria

Exclusion criteria for all participants were: (1) MRI contraindications, measured using the Monash Biomedical Imaging Magnetic Resonance Imaging (MRI) Screening and Information Form (*Supplementary Figure 1*); (2) unwillingness to refrain from any illicit substance and/or alcohol use in the 12 hours before testing (confirmed by self-report upon arrival at session); (3) current use of prescription medication that affects the central nervous system, except anti-depressants and anxiolytics due to prevalence of elevated depression/anxiety levels in CUD; (4) current suicidal ideation or history of any diagnosed psychiatric disorders, with the exception for depression and anxiety disorders due to the high comorbidity with CUD, as confirmed using The MINI International Neuropsychiatric Interview 7.0.2 (MINI; [1-4]; (5) history of any neurological disorders or major medical conditions (e.g., epilepsy, stroke, migraine, etc.); (6) history of acquired or traumatic brain injury or loss of consciousness > 5 minutes; (7) full scale intelligence quotient (FSIQ) estimate score < 70, confirmed using the Wechsler Abbreviated Scale of Intelligence – second edition (WASI-II; [5]; (8) current pregnancy and/or breastfeeding; (9) history of significant and regular mindfulness practice, defined as regular engagement in mindfulness practices over any extended period of time; or (10) significant dependence on alcohol, confirmed by a score > 19 on the Alcohol Use Disorders Identification Test (AUDIT; [6].

For people with a CUD only, exclusion criteria were: (1) significant dependence on or exposure to substances other than cannabis or tobacco, threshold at >50 occasions of use over a 2-year period in the past 10 years; or (2) or use of any illicit drug other than cannabis in the 4 weeks prior to testing.

For controls only, exclusion criteria were: (1) significant dependence on or exposure to substances other than tobacco, threshold at >50 occasions of use over a 2-year period in the past 10 years; (2) use of any illicit drug in the 4 weeks prior to testing; (3) use of cannabis at any stage in the 12 months prior to testing; or (4) > 50 lifetime uses of cannabis.

1. PROCEDURE

Members of the community who were interested in the study were directed to an online screening survey to determine their eligibility against inclusion and exclusion criteria (~30-minutes, 9,045 respondents). This was followed by a phone screener to confirm eligibility of potentially suitable participants (lasting ~10 minutes to ~1 hour; ~450 prospective people with a CUD and ~800 prospective controls were called). A detailed list of the tools used for the online and telephone screening is outlined in the study pre-registration (ISRCTN ID: 76056942). Eligible participants then attended face-to-face data collection at Monash Biomedical Imaging facility, Clayton, Victoria, Australia.

The testing session lasted approximately 4-6 hours (including the collection of measures beyond the scope of this study). Participants completed an assessment battery to profile socio-demographic data, substance use, mental health, and cognition, comprising questionnaires, face-to-face semi-structured interviews, standardised cognitive testing, as well as MRI scanning and provision of urine samples. All questionnaires used for participants’ screening and face-to-face testing were administered via Qualtrics Software, version 2019-2022 (Qualtrics, Provo, UT).

This study was part of a larger longitudinal study. At the end of testing, participants were offered the opportunity to debrief and receive reimbursement in the form of a Coles Myer voucher to the value of AUD $100 for controls, and of AUD $150 for people with a CUD, as they underwent additional testing to address research questions beyond the scope of this study. All participants were also offered a picture from a single frame from their T1-weighted (T1w) scan.

1. MEASURES

All measures were described in detail in the study pre-registration (<https://doi.org/10.1186/ISRCTN76056942>). Participants’ age, sex, and years of education were measured using a standard demographic proforma. Handedness was ascertained using the Edinburgh Handedness Inventory – Short Form (EHI-SF; [7]. The Wechsler Abbreviated Scale of Intelligence – second edition (WASI-II; [8]) was used to estimate participants’ FSIQ, derived from administering the Vocabulary and Matrix Reasoning subtests.

A diagnosis of at least moderate CUD was established using the SCID-5-RV [9]. Participants were required to respond ‘yes’ or ‘no’ to 11 items to determine the number of CUD endorsed. A score of 4-5 indicated moderate CUD, and a score of 6+ indicated severe CUD. Semi-structured interviews were administered to measure past-30-day-use of any substances (Timeline Follow Back [TLFB]; [10]; and lifetime cannabis use [11-13]. From these, days and dosage of cannabis, alcohol, and nicotine (i.e., grams, alcohol, and number of cigarettes; TLFB) were extracted, as well as age of regular cannabis use onset and duration of regular use, factoring in self-reported use breaks; regular use was defined as at least 3 x per week. Substance use and related problems were quantified for cannabis, alcohol, and nicotine using the CUDIT-R [14], the Alcohol Use Disorders Identification Test AUDIT; [15] and the Fagerström Test for Nicotine Dependence, respectively FTND; [16]. Urine levels of THC metabolites were also collected i.e., THC-COOH: creatinine ng/ml.

Several scales quantified mental health symptom scores. Depression was measured via the Beck Depression Inventory – second edition (BDI-II; [17]), state anxiety was assessed with the State-Trait Anxiety Index – Y Form, ‘state’ sub-scale STAI-Y; [18], and sub-clinical positive psychotic symptoms, negative psychotic symptoms and depressive symptoms were gauged with the Community Assessment of Psychic Experiences CAPE; [19]. Further, stress levels were measured using the Perceived Stress Scale PSS) – 10 items [20], while the COVID Stress Scale estimated COVID-specific stress [21].

1. fMRI ACQUISITION AND PROCESSING

**MRI Task Setup**

Prior to entering the scanner participants were requested to stay awake; the tester checked in real-time that participants kept their eyes open throughout the scan, via an MRI-compatible camera placed inside the MRI scanner.

**rsFC fMRI Task Instructions**

Inside the MRI scanner, and prior to the resting-state scan acquisition, participants were instructed verbatim by the researcher and via written instructions: “The next scan will take about 10 minutes. Keep your eyes open, try not to think about anything in particular. Stay relaxed and try to keep your head still”. Through the resting-state scan, participants were shown a fixation cross (white cross on black background) via a mirror placed inside the MRI scanner.

**MRI Data Acquisition Parameters**

Participants were scanned using the same group of experienced radiographers at the Monash Biomedical Imaging facility in Clayton, Victoria. All participants were scanned on the same Siemens Skyra 3 Tesla MRI scanner using a 32-channel head coil. *T1-weighted (T1w)* scans were acquired using magnetization-prepared rapid gradient-echo (MP-RAGE) sequence, with the following acquisition parameters: TE = 2.07ms, TR = 2300ms, flip angle = 9°, 192 sagittal slices without gap, field of view 256 x 256mm, yielding a 1 x 1 x 1mm resolution, for a total acquisition time of 5 minutes. *Resting-state* fMRI scans (189 volumes) using T2* weighted Echo Planar Imaging (EPI) were acquired using the following parameters: TE = 30ms, TR = 2500ms, flip angle = 90°, 44 slices without gap, field of view 192 x 192mm, matrix = 64, voxel size 3mm3, and a total acquisition time of 8 minutes. Slice based acceleration Generalised Autocalibrating Partially Parallel Acquisitions (GRAPPA) = 2 was applied. The Siemens system includes an additional two ‘dummy scans’ at the beginning of the acquisition to allow the magnetization to stabilise to a steady state. These two scans were not collected for any data analysis.

**MRI Data Handling**

All MRI data were directly exported from the scanner to Monash Biomedical Imaging-XNAT (XNAT website, private server), where it was stored and backed up in Digital Imaging and Communications in Medicine (DICOM) format. Raw format (i.e., DICOM) data were downloaded from the XNAT server and converted into Brain Imaging Data Structure (BIDS) format using dcm2niix (v1.0.20201102) for further analysis. All imaging data processing and analysis were performed on a cloud-based cluster-computational platform, MASSIVE (massive.org.au; Goscinski et al., 2014). The pre- and post-processing was conducted using CONN toolbox 20.b (www.nitrc.org/projects/conn, RRID:SCR_009550; Whitfield-Gabrieli & Nieto-Castanon, 2012), based on SPM12 on Matlab (2018a.r7487), which was pre-installed on MASSIVE.

**MRI Data Pre-Processing**

All validated data were imported in BIDS format, then underwent a standard pre-processing using CONN toolbox 20.b, including: (1) slice timing with interleaved slice order; (2) realignment and generation of motion parameters; (3) ARtifact-detection Tools (ART)-based outlier detection with intermediate settings (default 97^th^ percentile in normative sample); (4) co-registered fMRI data with T1w images; (5) segmentations of T1w images; (6) normalising T1w images to Montreal Neurological Institute (MNI) space (standard space) and normalising fMRI to MNI space with the same parameters; (7) default nuisance factors removal including six realignment parameters, six first order temporal derivatives, signal from white matter, cerebral spinal fluid, and whole brain, (8) smoothing with 6mm kernel; and (9) temporal bandpass filtering (0.008-0.09). Default CONN toolbox 20.b pre-processing steps also include a combination of aCompCor, scrubbing, and motion correction. fMRI was then resampled to 2 x 2 x 2mm isotropic voxels. Quality Assurance reports were generated and manually reviewed by authors HT and CS. Stringent criteria for detecting motion outliers, as outlined by Parkes et al. (2018), was followed, whereby limits of >0.25mm mean framewise displacement (mFD) and >5mm maximum framewise displacement were set; no participants violated these criteria.

**MRI Data Post-Processing**

The seed-based rsFC maps for the significant three seeds were used for further post-processing. The location of the seeds was determined by the default Harvard-Oxford atlas (132 cortical and subcortical regions; Desikan et al., 2006). Seed-based functional connectivity maps were generated using CONN toolbox 20.b. The group effect of the seed-based connectivity map was analysed using a General Linear Model (GLM) model using group as a factor (between subjects, two-group test). Covariates were age, sex, and total number of alcohol standard drinks over the past 30 days. Voxel wise multiple comparison error correction was applied, specifically a cluster-level False Discovery Rate (FDR) correction, p-corrected < 0.05 was applied, with default initial p < 0.001. To make the results more robust, a Benjamini-Hochberg procedure was additionally applied to alpha values to decrease the false discovery rate following the examination of multiple ROIs (Benjamini & Hochberg, 1995).

1. SUPPLEMENTARY TABLE 1.

**CUD group correlations between rsFC pairs showing group differences (rsFC beta values) correlated with metrics of cannabis exposure and related problems, depression scores, and nicotine dependency, using Bonferroni adjusted alpha threshold of p<.001 (.05 ÷ 45)**

| Seed region | Cluster region/s |  | | SCID-5-RV | CUDIT-R | Grams, past  30 days | THC-COOH: creatinine ng/ml | Hours since  last use | Age of  reg.  onset | Duration  of reg.  use | BDI-II^a^ | FTND |
| --- | --- | --- | --- | --- | --- | --- | --- | --- | --- | --- | --- | --- |
| *right*  NAc | frontal | r_s_ |  | -.10 | .03 | -.02 | -.03 | -.17 | .08 | .14 | .02 | -.13 |
|  |  | p |  | .45 | .83 | .89 | .83 | .19 | .54 | .28 | .88 | .30 |
| *left* putamen | occipito-parietal | r_s_ |  | .10 | -.10 | .04 | -.11 | .25 | -.28 | .08 | -.04 | -.11 |
|  |  | p |  | .45 | .46 | .73 | .38 | .05 | .02 | .53 | .78 | .41 |
| *left* pallidum | occipital | r_s_ |  | -.16 | -.42 | .12 | .11 | .13 | .01 | .02 | -.43 | -.10 |
|  |  | p |  | .20 | **<.001***** | .34 | .38 | .31 | .93 | .86 | **<.001***** | .44 |
| *right* pallidum | occipital | r_s_ |  | -.02 | .10 | -.02 | .10 | .18 | .10 | -.18 | -.08 | -.15 |
|  |  | p |  | .87 | .42 | .90 | .98 | .15 | .42 | .15 | .55 | .24 |
|  | occipital | r_s_ |  | .12 | -.05 | .08 | .04 | -.03 | .10 | -.14 | .05 | -.06 |
|  |  | p |  | .35 | .69 | .54 | .74 | .83 | .44 | .26 | .68 | .65 |

BDI-II, Beck Depression Inventory – second edition; CUDIT-R, Cannabis Use Disorder Identification Test-Revised; FTND, Fagerström Test for Nicotine Dependence; NAc, nucleus accumbens; r_s_, Spearman’s Rho; SCID-5-RV, Structured Clinical Interview of DSM-5 – research version; THC-COOH: creatinine ng/ml, 11-nor-9-carboxy-Δ⁹-tetrahydrocannabinol: creatinine per millilitre of urine

*Note*: Bolded p-values represent statistically significant, moderate (r_s_: 0.40 – 0.69) correlations *****<.001**

^a^ Due to collinearity with the BDI-II (Beck et al., 1996), CAPE (Stefanis et al., 2002) subscale scores were not included.

1. SUPPLEMENTARY FIGURE 1.

***Monash Biomedical Imaging Magnetic Resonance Imaging (MRI) Screening and Information Form***

Have you ever had any eye injury caused by metal? ...................................................................................NO / YES

If YES:

Did you see a doctor at the time? ............................................................................................................NO / YES

Did they remove the foreign body? .........................................................................................................NO / YES

Did they tell you that they got it all out? .................................................................................................NO / YES

Was this the last injury involving metal? ................................................................................................NO / YES

Are you pregnant, suspect you may be pregnant or breastfeeding?........................................ ....... .............NO / YES

**Do You Have (Or Have You Ever Had):**

A Cardiac Pacemaker/stent/defibrillator/wire..................................................................... ....... ................NO / YES

Any heart operation or valve replacement............................... ........................................... ....... ...............NO / YES

Any Brain operation ………………………………………………......................……………....... ..........NO / YES

Abdominal Aneurysm repair or IVC filter.................... ....... ......................................................................NO / YES

Brain Aneurysm Clips.................................................................... ....... .....................................................NO / YES

Deep Brain Stimulator....................................................................................... ....... .................................NO / YES

Brain Shunt Tube.................... ....... ............................................................................................................NO / YES

If YES, is it programmable ........................ ....... .........................................................................................NO / YES

Any Ear operations /cochlear or stapes implants.......... ....... ......................................................................NO / YES

Implanted drug infusion devices....................................................... ....... ..................................................NO / YES

Neuro or Bone growth stimulator......................................................................... ....... ..............................NO / YES

Shrapnel, bullet, gunshot............. ....... .......................................................................................................NO / YES

Any stents, vascular, oesophageal or biliary…............................................................................................NO / YES

Any Surgical clips/wire sutures/screws/mesh/prosthesis. ..........................................................................NO / YES

Joint Replacement or Prosthesis..................................................................................................................NO / YES

**Do You Have:**

Ocular prosthesis (eye implants)…....... ......................................................................................................NO / YES

A Swan-Ganz Catheter .................................. ....... ....................................................................................NO / YES

Skin patches .................................................................... ....... ...................................................................NO / YES

Intrauterine device (IUD)...................................................................... ....... ..............................................NO / YES

A penile prosthesis ................................................................................................ ....... .............................NO / YES

Any other implant, or breast tissue expander ....... .....................................................................................NO / YES

Tattooed eyelids or tattoos............................................. ....... ....................................................................NO / YES

Hearing Aid ....................................................................................... ....... ................................................NO / YES

Removable dentures............................................................... ....... ............................................................NO / YES

Any Piercings or braces that CANNOT be removed................................. ....... ........................................NO/ YES

Hair Extensions ………………………………………………………………………………....... ..........NO / YES

**Have You: What? / When?**

Had an operation or procedure within the last 8 weeks …………………………NO / YES .................................

Had a history of seizures or epilepsy .........................................................................................................NO / YES

References

1. Lecrubier, Y., et al., *The mini international neuropsychiatric interview.* European Psychiatry, 1998. **13**(1004): p. 198s-198s.

2. Lecrubier, Y., et al., *The Mini International Neuropsychiatric Interview (MINI). A short diagnostic structured interview: reliability and validity according to the CIDI.* European psychiatry, 1997. **12**(5): p. 224-231.

3. Sheehan, D., et al., *Mini international neuropsychiatric interview–version 7.0. 0 DSM-5. 2014.* Google Scholar, 2015.

4. Sheehan, D.V., et al., *The Mini-International Neuropsychiatric Interview (MINI): the development and validation of a structured diagnostic psychiatric interview for DSM-IV and ICD-10.* Journal of clinical psychiatry, 1998. **59**(20): p. 22-33.

5. Wechsler, D., *Wechsler Abbreviated Scale of Intelligence – Second Edition (WASI-II)*. 2011, San Antonio, TX: NCS Pearson.

6. Babor, T., et al., *AUDIT: the alcohol use disorders identification test: guidelines for use in primary health care. Geneva: World Health Organization; 2001.* BMC Public Health, 2018. **18**(1): p. 735.

7. Veale, J.F., *Edinburgh Handedness Inventory - Short Form: a revised version based on confirmatory factor analysis.* Laterality, 2014. **19**(2): p. 164-77.

8. Wechsler, D., *Wechsler Abbreviated Scale of Intelligence – Second Edition (WASI-II).* San Antonio, TX: Pearson. , 2011.

9. First, M.B., Williams, J. B., Karg, R. S., & Spitzer, R. L., *Structured clinical interview for DSM-5 – Research version (SCID-5-RV).* 2015.

10. Sobell, M.B., et al., *The reliability of a timeline method for assessing normal drinker college students' recent drinking history: utility for alcohol research.* Addict Behav, 1986. **11**(2): p. 149-61.

11. Lorenzetti, V., et al., *Gross morphological brain changes with chronic, heavy cannabis use.* Br J Psychiatry, 2015. **206**(1): p. 77-8.

12. Solowij, N., et al., *Cognitive functioning of long-term heavy cannabis users seeking treatment.* JAMA, 2002. **287**(9): p. 1123-31.

13. Yucel, M., et al., *Regional brain abnormalities associated with long-term heavy cannabis use.* Arch Gen Psychiatry, 2008. **65**(6): p. 694-701.

14. Adamson, S.J., et al., *An improved brief measure of cannabis misuse: the Cannabis Use Disorders Identification Test-Revised (CUDIT-R).* Drug Alcohol Depend, 2010. **110**(1-2): p. 137-43.

15. Babor, T., Higgins-Biddle, J., Saunders, J., & Monteiro, M. , *AUDIT: The Alcohol Use Disorders Identification Test: Guidelines for use in primary health care. Geneva: World Health Organization.* BMC Public Health, 2018. **18**(1): p. 735.

16. Fagerström, K., Russ, C., Yu, C. R., Yunis, C., & Foulds, J. , *The Fagerström Test for Nicotine Dependence as a predictor of smoking abstinence: A pooled analysis of varenicline clinical trial data.* Nicotine & Tobacco Research, 2012. **14**(12): p. 1467-1473.

17. Beck, A.T., Steer, R. A., & Brown, G., *Beck Depression Inventory–II.* Psychological Assessment. , 1996.

18. Spielberger, C., Gorsuch, R., Lushene, R., Vagg, P., & Jacobs, G. , *Manual for the state-trait anxiety scale.* Consulting Psychologists. , 1983.

19. Stefanis, N.C., Delespaul, P., Henquet, C., Bakoula, C., Stefanis, C. N., & Van Os, J. , *Early adolescent cannabis exposure and positive and negative dimensions of psychosis. .* Addiction, 2004. **99**(10): p. 1333–1341.

20. Cohen, S., Kamarck, T., & Mermelstein, R. , *A global measure of perceived stress. .* Journal of Health and Social Behavior, 1983. **14**(4): p. 385-396.

21. Taylor, S., et al., *Development and initial validation of the COVID Stress Scales.* J Anxiety Disord, 2020. **72**: p. 102232.
